# Atypical Reactive Center Kunitz-Type Inhibitor from the Sea Anemone *Heteractis crispa*

**DOI:** 10.3390/md10071545

**Published:** 2012-07-19

**Authors:** Irina Gladkikh, Margarita Monastyrnaya, Elena Leychenko, Elena Zelepuga, Victoria Chausova, Marina Isaeva, Stanislav Anastyuk, Yaroslav Andreev, Steve Peigneur, Jan Tytgat, Emma Kozlovkaya

**Affiliations:** 1 G.B. Elyakov Pacific Institute of Bioorganic Chemistry, Far Eastern Branch, Russian Academy of Sciences, 159, Pr. 100 let Vladivostoku, Vladivostok 690022, Russian Federation; Email: rita1950@mail.ru (M.M.); 969844@gmail.com (E.L.); zel@piboc.dvo.ru (E.Z.); v.chausova@gmail.com(V.C.); issaeva@gmail.com (M.I.); sanastyuk@piboc.dvo.ru (S.A.); kozempa@mail.ru (E.K.); 2 Shemyakin-Ovchinnikov Institute of Bioorganic Chemistry, Russian Academy of Sciences, 16/10, Miklukho-Maklaya Str., Moscow 117997, Russian Federation; Email: shifter2007@gmail.com; 3 Laboratory of Toxicology, University of Leuven (K.U. Leuven), Campus Gasthuisberg O&N2, Herestraat 49, P.O. Box 922, Leuven B-3000, Belgium; Email: Steve.Peigneur@pharm.kuleuven.be (S.P.); jan.tytgat@pharm.kuleuven.be (J.T.)

**Keywords:** sea anemone, Kunitz-type protease inhibitor, structure, function, channels

## Abstract

The primary structure of a new Kunitz-type protease inhibitor InhVJ from the sea anemone *Heteractis crispa* (*Radianthus macrodactylus*) was determined by protein sequencing and cDNA cloning. InhVJ amino acid sequence was shown to share high sequence identity (up to 98%) with the other known Kunitz-type sea anemones sequences. It was determined that the P1 Thr at the reactive site resulted in a decrease of the *K*_i_ of InhVJ to trypsin and α-chymotrypsin (7.38 × 10^−8^ M and 9.93 × 10^−7^ M, respectively). By structure modeling the functional importance of amino acids at the reactive site as well as at the weak contact site were determined. The significant role of Glu45 for the orientation and stabilization of the InhVJ-trypsin complex was elucidated. We can suggest that there has been an adaptive evolution of the P1 residue at the inhibitor reactive site providing specialization or functional diversification of the paralogs. The appearance of a key so-called P1 Thr residue instead of Lys might lead to refinement of inhibitor specificity in the direction of subfamilies of serine proteases. The absence of Kv channel and TRPV1-receptor modulation activity was confirmed by electrophysiological screening tests.

## 1. Introduction

Protein inhibitors regulate the activity of proteases involved in biochemical processes such as blood clotting, complement system, digestion, inflammation, apoptosis and others. In the case of protease inhibitor deficiency, some diseases may appear. Currently some attempts have been made in the therapeutic application of protease inhibitors [1,2,3]. Therefore, proteases and their inhibitors are attractive targets and intensively studied protein-protein complexes. Venomous animals are known to be a rich source of protease inhibitors, which can be used in biotechnology and medicine as prototypes for new generations of drugs. 

Besides neurotoxins, pore-forming toxins, and phospholipases A2, sea anemones also contain a large amount of protease inhibitors. To date about 40 low molecular mass protease inhibitors (6000 Da) from sea anemones have been isolated and partially characterized [4,5,6,7,8,9,10,11,12,13,14,15,16,17,18,19,20,21,22,23]. However, the complete amino acid sequences have only been determined for a few of them. The most known sea anemone inhibitors belong to the widely studied BPTI/Kunitz inhibitor family [24], except for Kasal-type inhibitors from Anemonia sulcata [13]. Their stable molecules consist of 56–60 amino acid residues, six of which are conservatively positioned cysteine residues forming three disulfide bonds. 

The Kunitz-type inhibitors interact with proteases by the classical substrate-like mechanism through a key P1 residue, which is located at the center of the canonical binding loop [25,26]. The majority of the sea anemone inhibitors have a positively charged Lys or Arg residue at the P1 position of the reactive site, which is known to be essential for inhibition of serine proteases, such as trypsin and α-chymotrypsin [11,17]. The inhibitor SHPI-1 from *Stichodactyla helianthus* has broad protease specificity and inhibits not only trypsin and chymotrypsin but also human neutrophil elastase (HNE), papain, and pepsin [11,27]. Some of the inhibitors from sea anemones are bifunctional molecules. They possess Kunitz-type protease as well as Kv1 channel inhibiting properties (type 2 toxins: AsKC1-AsKC3 or kalicludines 1–3 [17], SHTX III [20], and APEKTx1 [28]) or antihistamine activity (RmInI and RmInII [18]).

It is worth mentioning that the sea anemone Heteractis crispa (Radianthus macrodactylus) produces, besides P1 Lys/Arg inhibitors, atypical polypeptides with the Thr residue at P1 position of its reactive site. Jn-IV is shown to be an inhibitor of trypsin [15], and polypeptides APHC1-APHC3 weakly block the serine protease activity, but they modulate the activity of the TRPV1-receptor, causing the analgesic effect in experiments in vivo [21,22]. It has been well established that they also possess an anti-inflammatory activity [29]. Recently it has been deduced that the H. crispa polypeptides are encoded by a multigene superfamily and produced via a combinatory Kunitz-type library in the sea anemone venom [30]. According to the nature of a P1 residue of the 33 deduced mature polypeptides, three groups with Lys, Thr, and Arg have been categorized. It also has been suggested there has been an adaptive evolution of the P1 residue at the inhibitor reactive site providing specialization or functional diversification of the paralogs. 

In this paper we report the determination of the primary structure and structure-function features of a new inhibitor InhVJ from the sea anemone H. crispa. To elucidate the specificity of the InhVJ, an extensive screening on ion channels was also performed. Furthermore, the structures of serine protease complexes were also analyzed.

## 2. Results and Discussion

### 2.1. Purification and Primary Structure Determination of InhVJ

**Figure 1:**
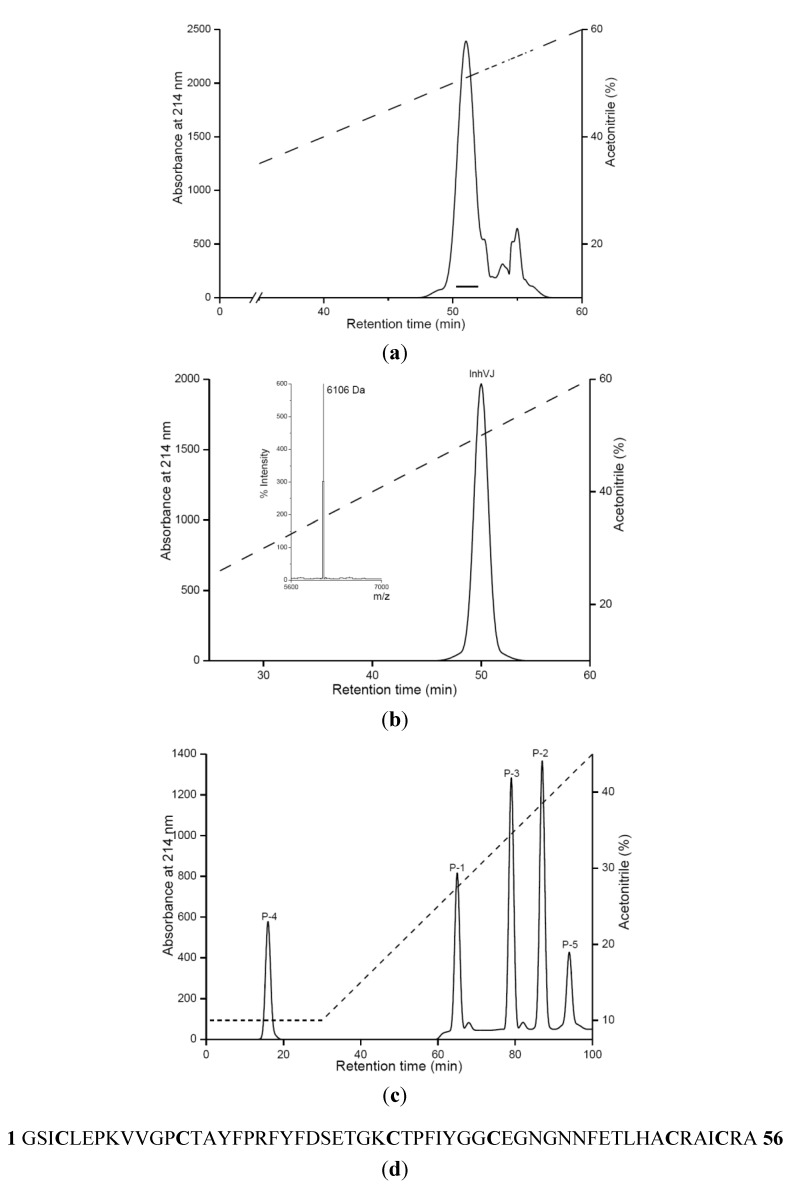
Purification of InhVJ, the result of alkylated inhibitor trypsin digestion and amino acid sequence of InhVJ. (**a**) RP-HPLC of fraction obtained after ion-exchange chromatography [19] on Nucleosil C_18_ (4.6 × 250 mm) column with a linear gradient of acetonitrile concentration; (**b**) Second purification of InhVJ by RP-HPLC under the same conditions described in A; the molecular mass of InhVJ determined by MALDI-TOF/MS (left inset of B); (**c**) RP-HPLC of peptides obtained after digestion of alkylated InhVJ by trypsin on Nucleosil C_18_ (4.6 × 250 mm) column with a linear gradient of acetonitrile concentration; (**d**) Amino acid sequence of InhVJ determined by Edman degradation.

The protease inhibitor InhVJ was isolated from 70%-ethanol extract of *H. crispa* (*R. macrodactylus*) by the scheme, which was proposed to separate inhibitors from polypeptides, such as neurotoxins, actinoporins, and phospholipases A2. This scheme was described previously [19]. In brief, it includes a hydrophobic chromatography on Polychrome-1, gel filtration chromatography on Bio-gel P-4 and ion-exchange chromatography on SP-Sephadex G-25. The obtained fractions were further purified by RP-HPLC on Nucleosil C_18_ column (Figure 1a). 

As a result of the second purification by RP-HPLC under the same conditions, the individual polypeptide was obtained and named InhVJ (Figure 1b). Its amino acid composition was characterized by the presence of six cysteine residues and the absence of tryptophan and methionine [19]. According to MALDI-TOF/MS data, the InhVJ molecular mass was 6106 Da. InhVJ showed both trypsin (*K*_i_ = 7.38 × 10^−8^ M) and α-chymotrypsin (*K*_i_ = 9.93 × 10^−7^ M) inhibiting activity [31].

To determine a complete amino acid sequence, alkylation of InhVJ and its further digestion by trypsin were carried out. The resulting five peptides obtained after the digestion were separated and desalted by RP-HPLC (Figure 1c). In Table 1, the molecular masses of each peptide determined by MALDI-TOF/MS and the amino acid sequences determined by Edman degradation are shown.

**Table 1:** Amino acid sequences and molecular masses of tryptic peptides P1–P5.

Peptide	Amino acid sequence	Molecular mass (MALDI)Dа	Molecular mass (theoretical)Da
P-1	GSICLEPK	951.31	951.01
P-2	VVGPCTAYFPR	1314.91	1314.42
P-3	FYFDSETGK	1094.54	1093.15
P-4	AICRA	637.73	637.66
P-5	CTPFIYGGCEGNGNNFETLHACR	2818.95	2818.75

On the basis of a tryptic peptide sequence comparison with the known sequence of the native inhibitor Jn-IV from H. crispa [15], the complete amino acid sequence of InhVJ (Figure 1d) was deduced. InhVJ consists of 56 amino acid residues and has a molecular mass according to the sequence analysis equal to 6112 Da. Since the molecular mass determined by MALDI-TOF/MS is 6106 Da, the difference of six mass units indicates that all six cysteine residues are involved in three disulfide bonds. 

**Figure 2:**
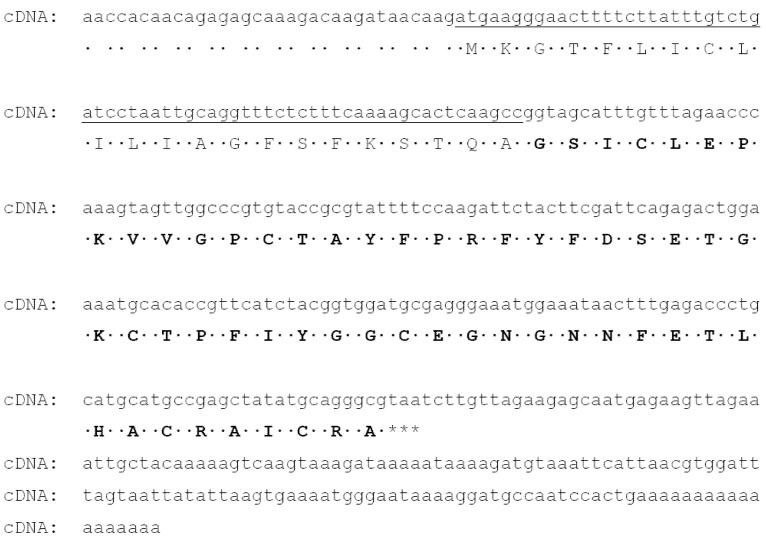
Nucleotide sequence of full-length cDNA encoding InhVJ. The signal sequence is underlined, the amino acid sequence of InhVJ is shown by bold types, the stop-codon is marked by asterisks.

The full-length cDNA encoding InhVJ was successfully obtained by combination of 3′ and 5′RACE techniques. It should be noted that the transcript corresponding to the full-length InhVJ was one of the most abundant transcripts (13.5%). The full-length cDNA includes 427 bp with an open reading frame of 234 bp, which encodes a polypeptide of 78 amino acid residues. The signal peptide is composed of 22 amino acid residues. The mature polypeptide (56 aa) is identical to the sequence obtained by Edman degradation (Figure 2). 

### 2.2. Amino Acid Sequences and Phylogenetic Analysis

**Figure 3:**
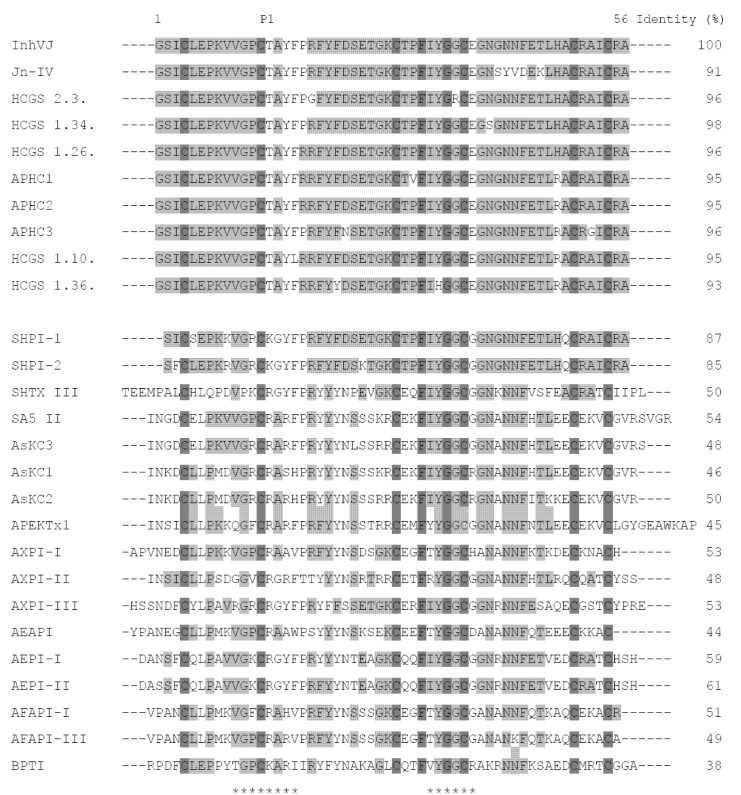
Alignment of amino acid sequences of protease inhibitors, analgesic polypeptides, Kv channels toxins from sea anemones, and protease inhibitor from bovine pancreas. InhVJ, Jn-IV, APHC1-APHC3, HCGS 2.3, 1.34, 1.26, 1.10, 1.36 from *H. crispa* [15,21,22,30]; SHPI-1, SHPI-2 from *S.helianthus* [11,12];SA5 II, AsKC1-AsKC3 from *A. sulcata* [16,17];SHTX III from *Stichodactyla haddoni* [20]; APEKTx1 from *Anthopleura elegantissima* [28]; AXPI-I, AXPI-II, and AXPI-III from *Anthopleura* aff. *xanthogrammica* [9,10]; AEAPI, AEPI-I, and AEPI-II from *Actiniaequina* [14,23]; AFAPI-I, AFAPI-III from *Anthopleurafuscoviridis* [23]; BPTI from *Bos taurus* [33]. Р1—amino acid residue of the inhibitor’s reactive center. The asterisks below the sequence of BPTI indicate the contact sites with serine proteases. Identical and conservative residues are indicated by dark and light gray colors.

A sequence similarity search in the GenBank database by the BLASTP program [32] revealed that the mature polypeptide InhVJ belongs to the Kunitz-type protease family. When aligned with the primary structures of known inhibitors (Figure 3), InhVJ shares the highest degree of homology—from 91 to 96% of identity—with the trypsin inhibitor Jn-IV and with analgesic polypeptides APHC1-APHC3 from *H.crispa*. It also shares a high degree of homology with HCGS-polypeptides recently deduced by the PCR-based cloning technique [30].

The sequence identity of InhVJ and inhibitors from other sea anemone species ranges from 46% to 87%. It is worth mentioning that protease inhibitors from sea anemones belonging to the same family have a higher degree of homology than the inhibitors from sea anemones belonging to different families. The percentage of identity of inhibitors from H. crispa (InhVJ), S. haddoni (SHTX III), and from S. helianthus (SHPI-1, SHPI-2) of the family Stichodactylidae is 50, 85 and 87%, respectively, whereas the percentage of identity of inhibitors from A. elegantissima (APEKTx1), A. aff. xanthogrammica (AXPI-I, AXPI-II, AXPI-III), A. sulcata (SA5 II, AsKC1-AsKC3), A. equina (AEAPI, AEPI-I, AEPI-II), and from A. fuscoviridis (AFAPI-I, AFAPI-III) of the family Actiniidae is in the range of 44%–61%. Besides, InhVJ shares a high degree of homology (up to 50%) with the polypeptides AsKC1-AsKC3, APEKTx1, and SHTX III, which also modulate voltage-gated potassium channel activity. Apparently, even though all these polypeptides evolved from a common ancestor and their structural Kunitz-fold has undergone hardly any significant changes during evolution, changes in variable parts of the molecules have taken place. These changes have not affected the overall fold of the polypeptide chain, but have led to the appearance of new functions such as Kv-channel inhibiting activity (type 2 toxins) or TRPV1-receptor modulating activity.

Recently it was deduced that the *H. crispa* polypeptides are encoded by a multigene superfamily and produced via a combinatory Kunitz-type library in the sea anemone venom [30]. Combinatorial libraries of polypeptides have also been found in other poisonous organisms, such as cone snails [34], spiders [35], and scorpions [36]. The 33 deduced mature GS-polypeptides were divided into three groups according to the P1 amino acid residue at the reactive site: group I—with Lys, group II—with Thr, group III—with Arg. So, InhVJ with the Thr residue at the reactive site belongs to structural group II. 

**Figure 4:**
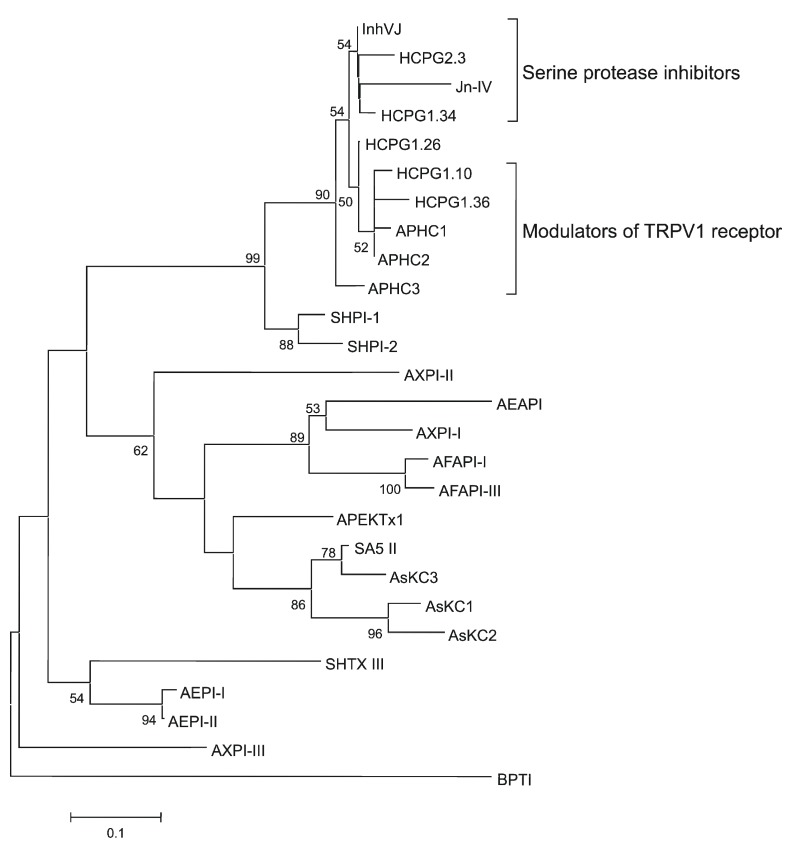
Evolutionary relationships of the known sea anemone Kunitz-type protease inhibitors. The NJ phylogenetic tree was made using the Poisson correction with bootstrap test of 1000 replications in MEGA 4 [37]. Nodes with confidence values greater than 50% are indicated.

The phylogenetic analysis was performed to determine evolutionary relationships of sea anemone serine protease inhibitors. According to the NJ phylogenetic tree, InhVJ as well as Jn-IV, APHC1-APHC3, SHPI-1, and SHPI-2 protease inhibitors of the Stichodactylidae family belong to one cluster, except for inhibitor SHTX III. However, one can see that here is an additional dividing of H. crispa polypeptides into two subsets in this cluster, InhVJ-like and APHC1-APHC3-like subsets (Figure 4). Previously we have shown that the additional dividing of these polypeptides is also confirmed by the surface electrostatic potential distribution [30]. The InhVJ-like subset includes molecules with neutral or slightly positive total charges ranging from +0.02 to +1.03, while the APHC1-APHC3-like subset contains molecules with more positive charges ranging from +1.81 to +2.15. We can suggest that there has been an adaptive evolution of the P1 residue at the inhibitor reactive site providing specialization or functional diversification of the paralogs. On the one hand, the appearance of the Thr P1 residue instead of Lys might lead to refinement of inhibitor specificity in the direction of subfamilies of serine proteases. On the other hand, it also might lead to the appearance of polypeptides with, for instance, a new function such as TRPV1 modulation.

### 2.3. Electrophysiological Experiments

**Figure 5:**
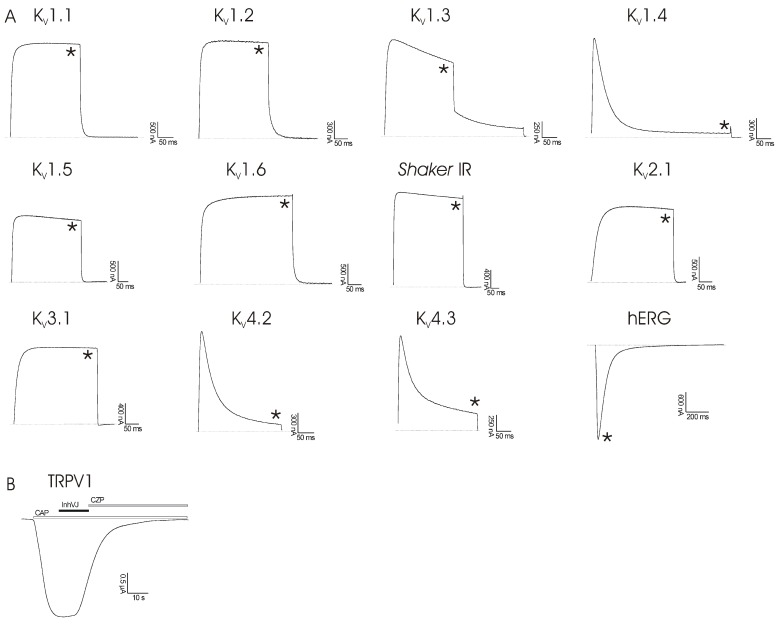
(**a**) Activity profile of InhVJ on several K_V_ channel isoforms and the cardiac hERG channel. The figure shows the results of one representative experiment. Whole-cell current traces in control and inhibitor conditions are shown. The dotted line indicates the zero-current level. The asterisk (*) marks steady-state current traces after application of 50 µM inhibitor. No differences in currents were observed between control and toxin situation. (**b**) Activity profile of InhVJ on TRPV1-receptor. No activity after application of 10 µM InhVJ was observed. CAP = capsaicine (2 μM); CZP = capsazepine (10 μM). All data represent the average of at least three independent experiments (*n* ≥ 3) and are presented as mean ± standard error.

Type 2 toxins from sea anemones are known to be potent inhibitors of voltage-gated potassium channels. Kalicludines block Kv1.2 channels with an IC50 value of about 1 µM [17], SHTX III inhibits the binding of known potassium channel ligands, like 125I-α-DTX with a Kd value of 650 nM [20], and APEKTx1 blocks Kv1.1 channels with an IC50 value of 0.9 nM [28]. Despite the high sequence identity of InhVJ with these polypeptides (up to 50%) and our previous suggestion, based on the molecular modeling results that show that InhVJ might also inhibit KV channels, electrophysiological screening tests on several KV channel isoforms (together with the cardiac hERG channel) revealed that InhVJ in a concentration range of up to 50 µM had no modulatory activity (Figure 5a). Kv channel expressing oocytes were, without pre-incubation, exposed to 50 µM InhVJ for a period of time ranging from 5 to 10 min. All experiments were performed on at least three different oocytes (n ≥ 3). No significant alteration of the potassium currents was observed (p < 0.05). It has been proposed that heavily charged polypeptides adapt more easily to the negative electrostatic potential of the extracellular part of the potassium channels [38]. Since the total charge of InhVJ is only slightly positive +0.02, it apparently is not enough to inhibit KV channels. But perhaps more importantly, there is also no functional dyad consisting of basic and hydrophobic (aromatic) amino acids in the structure of InhVJ. They were hypothesized as key residues for such activity in APEKTx1 [28]. 

As for InhVJ’s ability of blocking TRPV1-receptor activity, electrophysiological analysis revealed no activity of InhVJ in a concentration of up to 10 µM (Figure 5b). Oocytes expressing TRPV1 channels were, without pre-incubation, exposed to 10 µM of InhVJ in the presence of 2 µM capsaicine for time intervals ranging from 10 to 60 s. No significant effect was observed (p < 0.03). APHC1 was shown to modulate TRPV1-receptor with an EC50 value of about 54 nM and inhibit capsaicin-induced currents (32% ± 9%) [21]. InhVJ and APHC1-APHC3 shared 91% to 96% sequence identity. The main difference between the polypeptide sequences was the substitution of His48Arg, which is important for TRPV1 modulation activity [21,31,39]. So the experimental data is in complete agreement with the results of phylogenetic analysis, where dividing of a cluster into two subsets of InhVJ-like and analgesic polypeptides occurs. These results also confirm our suggestion that there is a functional specialization of InhVJ to only protease inhibition during evolution.

### 2.4. Comparison of Inhibitor Protease Inhibiting Activity

The best-studied polypeptide of the Kunitz family BPTI is a potent inhibitor of serine proteases (*K*_i_ in the range 10^11^–10^14^ M). BPTI Lys14 (the numbering is based on the sequence of InhVJ) P1 amino acid residue located at the center of the canonical binding loop plays an important role in the formation of stable complexes with proteases. The side chain of Lys14 penetrates deeply into the active site of trypsin and forms electrostatic interactions with its Asp189. It has been shown that the replacement of BPTI Lys14 by point mutations into other amino acid residues leads to an increase of association constant in complexes with trypsin-like proteases. It was deduced that P1 aromatic (Trp, Phe), large hydrophobic (Met, Leu) and polar (Gln, Asn, Ser, His) amino acid residues form stronger complexes with trypsin and chymotrypsin than small (Gly, Ala), branched (Thr, Val, Ile) and acidic side-chains (Asp, Glu) do [40]. 

Similar to BPTI, there are functionally important P1 Lys or Arg residues at reactive sites of the sea anemone inhibitors (Figure 3). Indeed, the polypeptides of the sea anemones *H. crispa*, *A. sulcata*, *Rhodactis rhodostoma*, *S. helianthus*, and *A. elegantissima*, like BPTI, are active towards trypsin (Table 2).

**Table 2:** The inhibition constants of protease inhibitors from sea anemones.

Sea anemone	Inhibitor	*К*_i_ (М)	
		Trypsin	α-chymotrypsin
*H. crispa*	InhVJ	7.38 × 10^−8^ [31]	9.93 × 10^−7^ [31]
	Jn-IV	9.6 × 10^−9^ [15]	n.d.
	АРНС1	1.0 × 10^−6^ [21]	5.0 × 10^−6^ [21]
	АРНС2	0.9 × 10^−6 ^ [22]	4.5 × 10^−6^ [22]
	АРНС3	5.0 × 10^−7^ [22]	7.0 × 10^−6^ [22]
*A. sulcata*	SA5 II	3.0 × 10^−10^ [5]	n.d.
	AsKC1-AsKC3	30 × 10^−9^ [17]	n.d.
*R. rhodostoma*	-	9.5 × 10^−10^ [6]	3.3 × 10^−8^ [6]
*A. elegantissima*	APEKTx1	124 × 10^−9^ [28]	n.d.
*S. helianthus*	SHPI-1	1.1 × 10^−10^ [11]	2.3 × 10^−9^ [11]
*B. taurus*	BPTI	6.0 × 10^−14^ [41]	1.8 × 10^−13^ [42]

n.d.—not determined.

It is worth mentioning that in the InhVJ, Jn-IV, and APHC1-APHC3 polypeptide structures from *H. crispa*, instead of Lys/Arg at position P1, there is a Thr residue previously not encountered in the Kunitz-type inhibitors reactive site. InhVJ was shown to be a specific inhibitor of two serine proteases, namely trypsin and α-chymotrypsin, and it had no activity on plasmin, thrombin, kallilrein, cysteine protease—papain, and aspartic protease—pepsin [31]. As the interaction between inhibitors with branched amino acid residues at the reactive site with trypsin-like proteases is known to be disadvantageous [40,43], an affinity of InhVJ, Jn-IV, and APHC1-APHC3 to trypsin, as opposed to BPTI and other sea anemone inhibitors, proved predictably lower (*K*_i_ 10^−6^–10^−9^ M) (Table 2). However, the constant value of InhVJ association with trypsin [31] was about two orders of magnitude higher, 1.36 × 10^7^ M^−1^, than the same constant of a mutant form of the BPTI with the replacement Lys14Thr at the reactive site, 2.9 × 10^5^ М^−1^ [40]. The contribution to the energy value of InhVJ association with trypsin is probably made both by amino acid residues at positions 10–17 located at the reactive site of the inhibitor and by the amino acid residues at positions 33–38 located at the weak contact site [44]. The constant values of InhVJ and mutant BPTI Lys14Thr association complexes with α-chymotrypsin appeared to be of the same order and were 1.01 × 10^6^ M^−1^ and 2.9 × 10^6^ M^−1^, respectively. 

Serine proteases (trypsin and elastase) are known to be involved in many inflammatory processes [45,46]. The elastase active site, as opposed to that of trypsin and α-chymotrypsin sites, is much smaller and better adapted to interact with branched (Thr, Val, Ile) amino acid residues at the P1 position [43,44]. Inhibitors selectively inhibiting serine protease action can potentially be used in the therapy with or the design of new anti-inflammatory medicines. According to the *in silico* investigation, Jn-IV with Thr at P1 position was hypothesized to inhibit human neutrophil elastase HNE [29]. Apparently InhVJ can also possess this activity. However, this hypothesis requires further experimental confirmation.

To elucidate the functional importance of amino acid residues in the complexes of InhVJ with serine proteases we used structural modeling methods.

### 2.5. Structure Modeling

**Figure 6:**
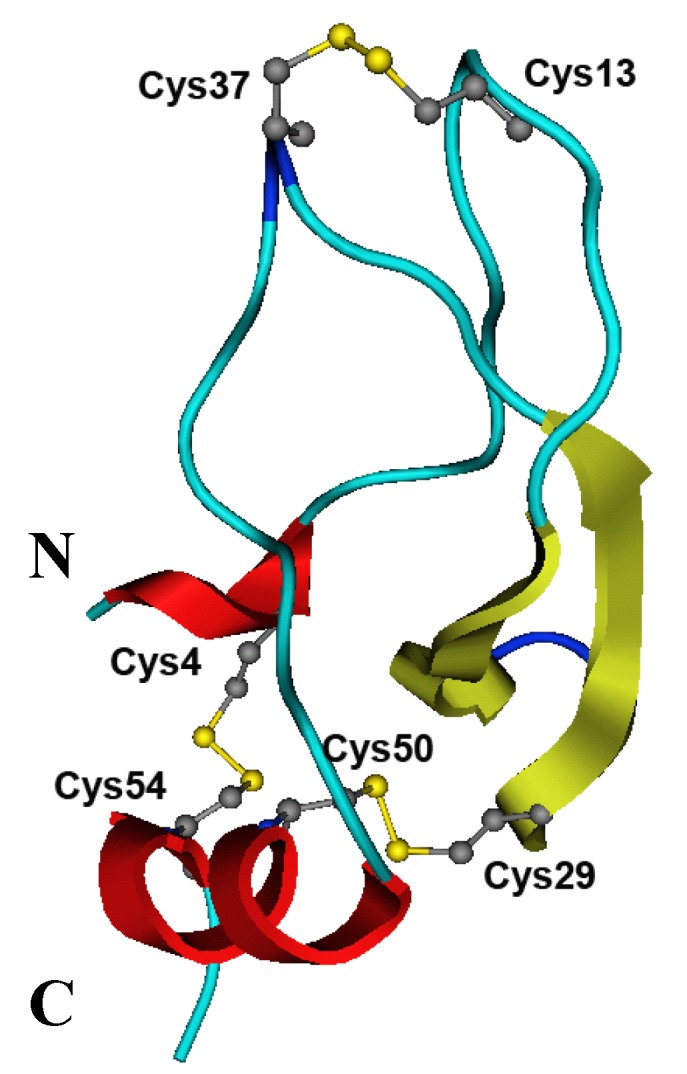
The structural model of InhVJ. The structure is shown as a ribbon diagram, where α-helixes are indicated by red color, β-strands by yellow, β-bends by dark blue, and disordered structure by blue colors. The positions of disulfide bonds in the structure of the inhibitor are indicated as dots of grey and yellow colors.

Currently, there is a unique spatial structure of the protease inhibitor from the sea anemone *S.helianthus* SHPI-1 (PDB 1SHP) established by ^1^H-NMR spectroscopy [47]. The high percentage of identity (87%) of InhVJ with SHPI-1 allowed us to use this inhibitor structure as a template. A structural model of InhVJ was generated using the server SWISS-MODEL [48,49,50] (Figure 6). After energy minimization using the MOE software with the capacity of the OPLS-AA [51], the energy of the molecule was −1987 kJ/mol.

To analyze the quality of the generated model, the RMSD parameter of the 55 Cα atoms InhVJ to those of SHPI-1 backbone traces was calculated (0.14 Å). The Ramachandran plots of φ, ψ angles [52] show that almost 75% of the non-glycine residues fall within the most favorable regions of the plot (data not shown). Remaining residues lie within the additional favorable regions as defined by PROCHECK [53].

The content of secondary structure elements in the InhVJ structural model was determined by Molmol [54]. The 3D-model shares a Kunitz scaffold as expected according to its high sequence identity with the template. The InhVJ structure like BPTI and SHPI-1 structures contains two α-helices, located at the *N*- and *C*-termini of the molecule, two antiparallel β-strands and two large loops (disordered structure) and is stabilized by three conservative disulfide bonds. Thus, the InhVJ structural model was accurate enough for further structural and functional studies.

**Figure 7:**
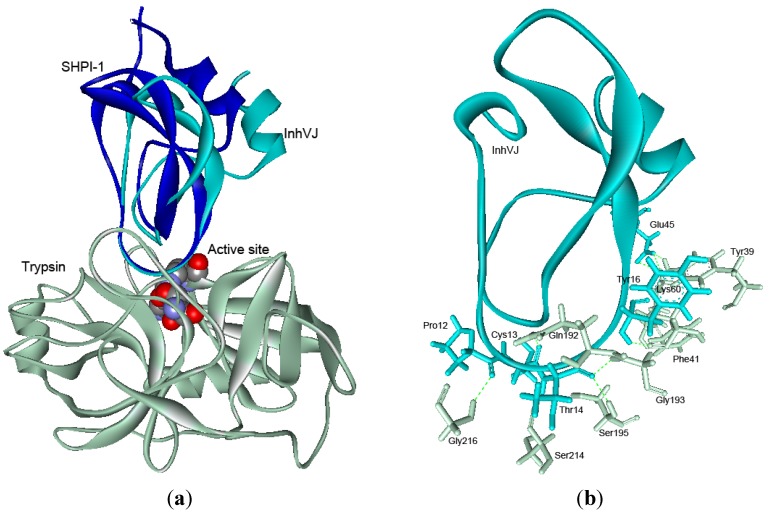
(**a**) Superimposition of the theoretical model of complex InhVJ-trypsin and crystal structure of complex SHPI-1-trypsin (PDB ID 3M7Q). Structures are shown as the ribbon diagrams. The key residues of the enzyme active site are shown as bowls. (**b**) Intermolecular hydrogen bonds in InhVJ-trypsin complex and interacting amino acid residues are shown as sticks for InhVJ in cyan and for trypsin in light gray colors.

To highlight the mechanism of InhVJ interaction with serine proteases by the method of molecular docking, a structural model of InhVJ-trypsin complex was designed (Figure 7a). According to the obtained data, the interaction of InhVJ with both trypsin and α-chymotrypsin (data is not shown) had a canonical character which is typical for the Kunitz-type polypeptides [55]. Docking analysis showed that InhVJ had a single binding site with the trypsin active site area, substantially overlapping with the SHPI-1 binding site (PDBID 3M7Q) [56]. Notably, superimposition of the structures of InhVJ and SHPI-1 complexes with trypsin also revealed that despite keeping the position of the InhVJ binding site with trypsin, there was a slight deviation of the InhVJ molecule with RMSD 3.2 Å.

Analysis of the polypeptide chain interactions in complex performed with MOE software shows that the complex formation is governed by van der Waals, hydrophobic and electrostatic forces as well as H-bonding (Figure 7b) (Supplementary data). One can see that the stabilizing H-bond number decreases in the complexes BPTI-trypsin > BPTI Lys14Thr-trypsin > InhVJ-trypsin > SHPI-1-trypsin. It is worth mentioning that the H-bond number formed by the P1 residue of inhibitors with the trypsin active site residues decreases from seven for BPTI Lys14 to five for SHPI-1 Lys14 and to three for InhVJ Thr14. These findings correlate well with the decreasing *K*_i_ values of their complexes (Table 2).

Besides, the complex interface analysis and the contribution of amino acid residues, localized at the reactive site (five amino acids) as well as at the weak contact site (three amino acids) to free binding energies for the complexes formation, were performed. According to computational data, the P1 Lys residue at the reactive site made the greatest contribution to the binding free energy for the complex formation. Whereas the contribution of the Thr residue at this position was reduced by almost half, but still quite substantial for both InhVJ and for the mutant BPTI Lys14Thr (Table 3). 

**Table 3:** The amino acid residues’ contribution to binding free energies and interfaces for the complexes of inhibitors with trypsin. Amino acid residues making the greatest contribution to binding free energy are highlighted in light green.

	Reactive site					Weak contact site		
**BPTI (3OTJ)**	**Pro12**	**Cys13**	**Lys14**	**Ala15**	**Arg16**	**Cys37**	**Arg38**	**Lys45**
ΔG_bind_, Kcal/Mol	−2.1	−4.8	−8.1	−3.3	−3.2	−1.8	−2	1.1
ΔSASA, Ǻ^2^	33	51.6	165.2	36	110.8	27.2	97.2	0
**BPTI mutant (3BTT)**	**Pro12**	**Cys13**	**Thr14**	**Ala15**	**Arg16**	**Cys37**	**Arg38**	**Lys45**
ΔG_bind_, Kcal/Mol	−2.1	−4,6	−4.4	−3.5	−3.5	−1.7	−1.6	0.3
ΔSASA, Ǻ^2^	37.9	55.5	101.4	36.5	118.3	28.6	94.6	0
**SHPI-1 (3M7Q)**	**Arg12**	**Cys13**	**Lys14**	**Gly15**	**Tyr16**	**Cys37**	**Gly38**	**Glu45**
ΔG_bind_, Kcal/Mol	−3.4	−2.9	−8.4	−2.2	−4.5	−1.4	−0.1	−0.5
ΔSASA, Ǻ^2^	143.8	52	163.6	19.9	88.7	30	0.9	0
**InhVJ**	**Pro12**	**Cys13**	**Thr14**	**Ala15**	**Tyr16**	**Cys37**	**Glu38**	**Glu45**
ΔG_bind_, Kcal/Mol	−2.1	−4.4	−4.7	−2.6	−5.9	−1.1	−0.2	−8.4
ΔSASA, Ǻ^2^	50.5	53	106.1	30.5	102.2	37.2	0	37.9

It should be noted that the presence of Tyr16 in InhVJ instead of Arg16 in BPTI was more advantageous in terms of solvation energy. One can see the decrease of the binding free energy from −3.2 Kcal/Mol for the BPTI-trypsin complex to −5.9 Kcal/Mol for the InhVJ-trypsin complex. However, this advantage was not enough to compensate the energy loss caused by the Lys to Thr replacement. In this case there was the electrostatic potential weakening in the area of the InhVJ reactive site, which apparently resulted in the decrease of the *K*_i_ value of the InhVJ-trypsin complex. 

**Figure 8:**
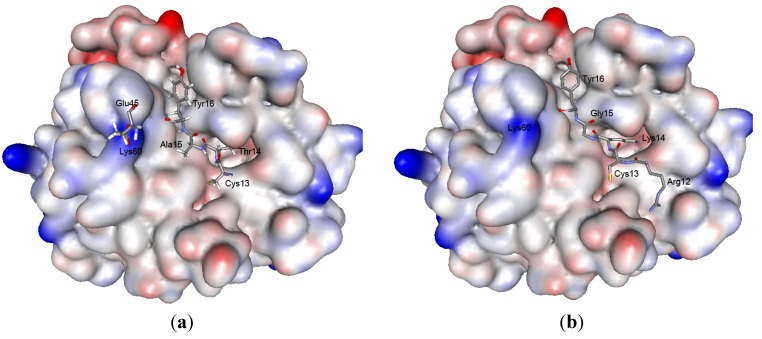
«Anchor» residues with maximum binding free energies contribution upon formation complexes of InhVJ (**a**) and SHPI-1 (**b**) with trypsin. Trypsin is shown as a solvent accessible surface colored by atomic partial charges (red is negative, blue is positive). Inhibitors «anchor» residues represented as sticks.

At the weak contact site of inhibitors, two residues make the main energy gain. BPTI Arg38 forms two hydrogen bonds with the Asn97 of trypsin (supplementary data), and contributes to the binding free energy by about −2.0 Kcal/Mol. At this position in the InhVJ molecule there occurs a negative charge (Glu38), which does not influence the complex binding free energy. At the same time, in the InhVJ-trypsin complex, Glu45 makes the most substantial negative contribution to the complex formation energy (−8.4 Kcal/Mol), unlike BPTI Lys45 with a positive contribution. The strong electrostatic attraction of Glu45 to Lys60 (Figure 8) of the enzyme summing up with the hydrogen bond formation with Lys60 as well as with Tyr39 (Supplementary data) apparently determines the orientation and additional stabilization of InhVJ in the complex. These facts indicate the enhancement of the weak contact site role, in particular the Glu45 residue, in the formation of a InhVJ-trypsin complex, while the role of the reactive site residues, in particular Thr14 and Tyr16, in our opinion might be the stabilization of the complex through the formation of hydrophobic interactions and hydrogen bonding networks. According to computational data, a similar regularity of the role of enhanced weak contact site residues was observed for the InhVJ-α-chymotrypsin complex (data is not shown).

## 3. Experimental Section

### 3.1. Purification of InhVJ

Specimens of the sea anemone *H. crispa* (*R. macrodactylus*) were collected in the coral reefs of the Seychelles during a marine expedition aboard the research vessel “Academik Oparin”. Dr. C. D. Grybelniy (Zoological Institute of the Russian Academy of Sciences, Saint-Petersburg, Russia) confirmed the identity of the species. InhVJ was isolated from 70%-ethanol extract of *H. crispa* by the scheme, which included a hydrophobic chromatography on Polychrome-1, gel filtration chromatography on Bio-gel P-4 and ion-exchange chromatography on SP-Sephadex G-25 [19]. The obtained fractions were purified by RP-HPLC on Nucleosil C_18_ column (4.6 × 250 mm; Sigma Aldrich, USA) with monitoring at 214 nm. Solvent A was 0.1% trifluoroacetic acid (TFA) in water, solvent B was 0.1% TFA in acetonitrile. A linear gradient from 10% to 60% of solvent B concentration was performed at a flow rate of 0.5 mL/min for 60 min. As the result of the second purification by RP-HPLC under the same conditions the individual polypeptide was obtained.

### 3.2. Chemical and Enzymatic Treatment

InhVJ was reduced and alkylated with 4-vinylpyridine as described in [19]. The resulting product was purified by RP-HPLC on a Nucleosil C_18_ column (4.6 × 250 mm; Sigma Aldrich, USA) with monitoring at 214 nm. Solvent A was 0.1% TFA in water, solvent B was 0.1% TFA in acetonitrile. A linear gradient from 10 to 60% of solvent B concentration was performed at a flow rate of 0.5 mL/min for 100 min. Alkylated polypeptide was digested with trypsin (Sigma Aldrich, USA) 1:50 (w/w) in 300 µL of 100 mМ ammonium bicarbonate (рН 8.0) at 37 °С for 24 h. Tryptic peptides were separated and desalted by RP-HPLC. 

### 3.3. Physical-Chemical Characterization

Molecular weight determination of InhVJ and tryptic peptides was performed by matrix-assisted laser desorption ionization time-of-flight mass spectrometry (MALDI-TOF/MS) in linear positive ion mode, using BIFLEX-III instrument (Bruker Daltonic, Germany). Sinapinic acid was used as a matrix. Angiotensin-II (Sigma, USA) and matrix peaks were used for calibration of the instrument. The amino acid sequences of tryptic peptides were determined on an automated sequencer protein «Procise 492 cLC» (Applied Biosystems, USA).

### 3.4. RACE Techniques, Cloning Experiments and Sequencing

The full-length cDNA of InhVJ was performed by 3′ and 5′RACE technique with *H. crispa* tentacles cDNA synthesized previously [57]. The nucleotide sequences of the primers and PCR conditions were the same as described in [30]. Briefly, the first 3′RACE reaction was performed with a degenerate Inh 1 primer and the second 3′RACE one did with a degenerate Inh 2 primer. On the basis of the determined partial 3′RACE sequences, the remaining 5′-terminal sequences were obtained using three gene-specific primers: RmIn-1-5 (step 1), RmIn-AVJ (step 2), and RmIn-3-5AVJ (step 3).

The 3′RACE (~300 bp) and the 5′RACE (~200 bp) PCR fragments were gel separated, purified and TA cloned (Fermentas, UAB) into *Escherichiacoli* Top10 strain (Invitrogen, USA). Plasmids were sequenced by the dideoxy chain termination method using the ABI PRISM 3130xL Genetic Analyzer (Applied Biosystems, USA). cDNA sequences were analyzed applying the CromasPro (Applied Biosystems, USA) and Vector NTI (Invitrogen, USA) programs. 

### 3.5. Phylogenetic Analysis

Searching homology sequences analysis was carried out using protein databases and BLAST programs [32]. Phylogenetic analysis of the known sea anemone Kunitz-type protease inhibitors was performed using the neighbor-joining (NJ) method with Poisson correction [58,59] in the MEGA 4 [37]. The bootstrap was performed with 1000 replicates. BPTI was used as an out-group. 

### 3.6. Expression of Voltage-Gated Ion Channels in Xenopus Laevis Oocytes

For the expression of voltage-gated potassium channels (rK_V_1.1, rK_V_1.2, hK_V_1.3, rK_V_1.4, rK_V_1.5, rK_V_1.6, Shaker IR, rK_V_2.1, hK_V_3.1, rK_V_4.2, rK_V_4.3, hERG), and the capsaicine receptor (TRPV1) in *Xenopuslaevis* oocytes, the linearized plasmids were transcribed using the T7 or SP6 mMESSAGE-mMACHINE transcription kit (Ambion, USA). The harvesting of stage V-VI oocytes from anaesthetized female *X. laevis* frogs was previously described [60]. Oocytes were injected with 50 nL of cRNA at a concentration of 1 ng/nL using a micro-injector (Drummond Scientific, USA). The oocytes were incubated in a solution containing (in mM): NaCl, 96; KCl, 2; CaCl_2_, 1.8; MgCl_2_, 2 and HEPES, 5 (pH 7.4), supplemented with 50 mg/L gentamycin sulfate.

### 3.7. Electrophysiological Recordings

Two-electrode voltage-clamp recordings were performed at room temperature (18–22 °C) using a Geneclamp 500 amplifier (Molecular Devices, USA) controlled by a pClamp data acquisition system (Axon Instruments, USA). Whole cell currents from oocytes were recorded 1–4 days after injection. The bath solution composition was ND96 (in mM): NaCl, 96; KCl, 2; CaCl_2_, 1.8; MgCl_2_, 2 and HEPES, 5 (pH 7.4) or HK (in mM): NaCl, 2; KCl, 96; CaCl_2_, 1.8; MgCl_2_, 2 and HEPES, 5 (pH 7.4). Voltage and current electrodes were filled with 3 M KCl. Resistances of both electrodes were kept between 0.5 and 1.5 MΩ. The elicited currents were filtered and sampled at resp. 1 kHz and 10 kHz (for potassium currents) and at resp. 0.2 kHz and 0.5 kHz (for TRPV1 currents), using a four-pole low-pass Bessel filter. Leak subtraction was performed using a P/4 protocol. K_V_1.1-K_V_1.6 and *Shaker* currents were evoked by 500 ms depolarizations to 0 mV followed by a 500 ms pulse to −50 mV, from a holding potential of −90 mV. Current traces of hERG channels were elicited by applying a +40 mV prepulse for 2 s followed by a step to −120 mV for 2 s. K_V_2.1, K_V_3.1 and K_V_4.2, K_V_4.3 currents were elicited by 500 ms pulses to +20 mV from a holding potential of −90 mV. TRPV1 currents were measured in ND96 solution using a protocol of −90 mV during 400 s. The recording chamber was perfused at a rate of 2 mL min^−1^ with a ND-96 solution containing (in mM) 96 NaCl, 2 KCl, 1.8 CaCl_2_, 1 MgCl_2_, 5 HEPES, pH 7.4. As previously described [61], capsaicin (2 μM) was used as an agonist and capsazepine (10 μM) as an antagonist of TRPV1. Capsaicin and capsazepine were purchased from Sigma. All data represent at least three independent experiments (*n* ≥ 3) and are presented as mean ± standard error. 

### 3.8. Homology Modeling and Docking

A InhVJ spatial structure model was generated by homology modeling method using SWISS-MODEL server [49] with the Kunitz type sea anemone polypeptide SHPI-1 crystal structure (PDB ID 1SHP) as a template [47]. Generated models were energy minimized using the MOE software with the capacity of the OPLS-AA [51]. Stereochemical quality was checked using Ramachandran plot and PROCHECK server [52,53]. 

The structural models of the InhVJ-trypsin as well as InhVJ-α-chymotrypsin complexes were generated using the molecular docking method by the ClusPro2.0 server resources [62]. Crystal structure of trypsin and α-chymotrypsin were extracted from 2PTC [63] and 1CBW [64] PDB files, respectively. Analysis of molecular interface and amino acid residue contribution to complex stabilization were performed using webservers ANCHOR [65,66] and PDBe PISA [67]. “Anchor” residues was identified as five amino acid residues of those that showed the maximum contribution to the predicted binding free energy and change in solvent accessible surface area (ΔSASA) for enzyme-inhibitor complexes [68]. Visualizations were performed using DS Visualizer 2.5 Accelrys^®^ software.

## 4. Conclusions

Sea anemones are known to produce a large amount of Kunitz-type polypeptides, which can possess the feature of a protease inhibitor as well as of a modulator of Kv or TRPV1 channels. InhVJ, a new Kunitz-type inhibitor with the so-called P1 Thr residue is specific to trypsin-like proteases and does not modulate Kv or TRPV1 channels. This inhibitor forms a stable complex with trypsin and is stabilized by H-bonds of the P1 Thr residue at the reactive site with the protease active site residues, as well as via electrostatic interaction and H-bonds of Glu45 at the weak contact site with protease Lys60 and Tyr39.

## References

[1] Kristeller J.L., Roslund B.P., Stahl R.F. (2008). Benefits and risks of aprotinin use during cardiac surgery. Pharmacotherapy.

[2] Zhou L.W., Wang Y.L., Yan X.T., He X.H. (2008). Urinary trypsin inhibitor treatment ameliorates acute lung and liver injury resulting from sepsis in a rat model. Saudi Med. J..

[3] Lin Y.F., Zhang N., Guo H.S., Kong D.S., Jiang T., Liang W., Zhao Z.H., Tang Q.Q., Ma D. (2007). Recombinant tissue factor pathway inhibitor induces apoptosis in cultured rat mesangial cells via its Kunitz-3 domain and *C*-terminal through inhibiting PI3-kinase/Akt pathway. Apoptosis.

[4] Fritz H., Brey B., Beress L. (1972). Polyvalent isoinhibitors of trypsin, chymotrypsin, plasmin and kallikrein from sea anemone (*Anemonia sulcata*), isolation, inhibition and amino acid composition. Hoppe Seyler Z. Physiol. Chem..

[5] Wunderer G., Beress L., Machleidt W., Fritz H., Lorand L. (1976). Broad Specificity. Inhibitors from Sea Anemones. Methods in Enzymology.

[6] Mebs D., Liebrich M., Reul A., Samejima Y. (1983). Hemolysins and proteinase inhibitors from sea anemones of the Gulf of Aqaba. Toxicon.

[7] Shiomi K., Ishikawa M., Yamanaka H., Kikuchi T. (1989). Isolation and properties of four serine protease inhibitors from water extracts of sea anemone *Actinia equine*. Nippon Suisan Gakkaishi.

[8] Mebs D., Gebauer E. (1980). Isolation of proteinase inhibitory, toxic and hemolytic polypeptides from the sea anemone *Stichodactyla* sp. Toxicon.

[9] Minagawa S., Ishida M., Shimakura K., Nagashima Y., Shiomi K. (1997). Isolation and amino acid sequences of two Kunitz-type protease inhibitors from the sea anemone *Anthopleura* aff. xanthogrammica. Comp. Biochem. Physiol. B Biochem. Mol. Biol..

[10] Minagawa S., Ishida M., Shimakura K., Nagashima Y., Shiomi K. (1998). Amino acid sequence and biological activities of another Kunitz-type protease inhibitor from the sea anemone *Anthopleura* aff. xanthogrammica. Fish Sci..

[11] Delfín J., Martínez I., Antuch W., Morera V., González Y., Rodríguez R., Márquez M., Saroyán A., Larionova N., Díaz J. (1996). Purification, characterization and immobilization of proteinase inhibitors from *Stichodactyla helianthus.*. Toxicon.

[12] Díaz J., Morera V., Delfín J., Huerta V., Lima G., Rodriguex de la Vega M., Garcia B., Padrón G., Assfalg-Machleidt I., Machleidt W. (1998). Purification and partial characterization of a novel proteinase inhibitor from the sea anemone *Stichodactyla helianthus*. Toxicon.

[13] Kolkenbrock H., Tschesche H.A. (1987). New inhibitor of elastase from the sea anemone *Anemonia sulcata.*. Biol. Chem. Hoppe Seyler.

[14] Ishida M., Minagawa S., Miyauchi K., Shimakura K., Nagashima Y., Shiomi K. (1997). Amino acid sequences of Kunitz-type protease inhibitors from the sea anemone *Actinia equine.*. Fish Sci..

[15] Zykova T.A., Vinokurov L.M., Markova L.F., Kozlovskaya E.P., Elyakov G.B. (1985). Amino-acid sequence of trypsin inhibitor IV from *Radianthus macrodactylus.*. Bioorg. Khim..

[16] Wunderer G., Machleidt W., Fritz H., Lorand L. (1981). The Broad-Specificity Proteinase Inhibitor 5 II from the Sea Anemone *Anemonia sulcata*. Methods in Enzymology.

[17] Schweitz H., Bruhn T., Guillemar E., Moinier D., Lancelin J.M., Béress L., Lazdunski M. (1995). Kalicludines and Kaliseptine: Two different classes of sea anemone toxins for voltage sensitive K^+^ cannels. J. Biol. Chem..

[18] Sokotun I.N., Il’ina A.P., Monastyrnaya M.M., Leychenko E.V., Es’kov A.A., Anastuk S.D., Kozlovskaya E.P. (2007). Proteinase inhibitors from the tropical sea anemone *Radianthus macrodactylus*: Isolation and characteristic. Biochemistry.

[19] Sokotun I.N., Leichenko E.V., Vakorina T.I., Es’kov A.A., Il’ina A.P., Monastyrnaia M.M., Kozlovskaia E.P. (2007). A serine protease inhibitor from the anemone *Radianthus macrodactylus*: Isolation and physicochemical characteristics. Bioorg. Khim..

[20] Honma T., Kawahata S., Ishida M., Nagai H., Nagashima Y., Shiomi K. (2008). Novel peptide toxins from the sea anemone *Stichodactyla haddoni.*. Peptides.

[21] Andreev Y.A., Kozlov S.A., Koshelev S.G., Ivanova E.A., Monastyrnaya M.M., Kozlovskaya E.P., Grishin E.V. (2008). Analgesic compound from sea anemone *Heteractis crispa* is the first polypeptide inhibitor of vanilloid receptor 1 (TRPV1). J. Biol. Chem..

[22] Kozlov S.A., Andreev Y.A., Murashev A.N., Skobtsov D.I., D’yachenko I.A., Grishin E.V. (2009). New polypeptide components from the *Heteractis crispa* sea anemone with analgesic activity. Bioorg. Khim..

[23] Minagawa S., Sugiyama M., Ishida M., Nagashima Y., Shiomi K. (2008). Kunitz-type protease inhibitors from acrorhagi of three species of sea anemones. Comp. Biochem. Physiol. B Biochem. Mol. Biol..

[24] Kunitz M., Northrop J. (1936). Isolation from beef pancreas of crystalline trypsinogen, trypsin, a trypsin inhibitor and intibular trypsin compound. J. Gen. Physiol..

[25] Bode W., Huger R. (2000). Structural basis of the endoproteinase-protein inhibitor interaction. Biochim. Biophys. Acta Protein Struct. Mol. Enzymol..

[26] Krowarsch D., Cierpicki T., Jelen F., Otlewski J. (2003). Canonical protein inhibitors of serine proteases. Cell Mol. Life Sci..

[27] Gil D.F., García-Fernández R., Alonso-del-Rivero M., Lamazares E., Pérez M., Varas L., Díaz J., Chávez M.A., González-González Y., Mansur M. (2011). Recombinant expression of ShPI-1A, a non-specific BPTI-Kunitz-type inhibitor, and its protection effect on proteolytic degradation of recombinant human miniproinsulin expressed in *Pichia pastoris*. FEMS Yeast Res..

[28] Peigneur S., Billen B., Derua R., Waelkens E., Debaveye S., Béress L., Tytgat J.  (2011). A bifunctional sea anemone peptide with Kunitz type protease and potassium channel inhibiting properties. Biochem. Pharmacol..

[29] Zelepuga E., Tabakmakher V., Monastyrnaya M., Lukyanov P., Kozlovskaya E. *In Silico* Investigation of Interaction between Human Neutrophil Elastase and Sea Anemone *Heteractis crispa* Kunitz Polypeptide. Proceedings of the 5th International Conference on Bioinformatics and Biomedical Engineering (ICBBE 2011).

[30] Isaeva M.P., Chausova V.E., Zelepuga E.A., Guzev K.V., Tabakmakher V.M., Monastyrnaya M.M., Kozlovskaya E.P. (2012). A new multigene superfamily of Kunitz-type protease inhibitors from sea anemone *Heteractis crispa.*. Peptides.

[31] Sokotun I.N., Gnedenko O.V., Leychenko E.V., Monastyrnaya M.M., Kozlovskaya E.P., Molnar A.A., Ivanov A.S. (2007). Study of the interaction of trypsin inhibitor from the sea anemone *Radianthus macrodactylus* with proteases. Biochem. (Mosc.) Suppl. Ser. B Biomed. Chem..

[32] Altschul S.F., Gish W., Miller W., Myers E.W., Lipman D.J. (1990). Basic local alignment search tool. J. Mol. Biol..

[33] Kassell B., Lorand L. (1970). Trypsin-Kallikrein Inhibitor (Kunitz Inhibitor, Basic Pancreatic Trypsin Inhibitor, Polyvalent Inhibitor from Bovine Organs). Methods in Enzymology.

[34] Olivera B.M., Hillyard D.R., Marsh M., Yoshikami D. (1995). Combinatorial peptide libraries in drug design: Lessons from venomous cone snails. Trends Biotechnol..

[35] Sollod B.L., Wilson D., Zhaxybayeva O., Gogarten J.P., Drinkwater R., King G.F. (2005). Were arachnids the first to use combinatorial peptide libraries?. Peptides.

[36] Kozminsky-Atias A., Bar-Shalom A., Mishmar D., Zilberberg N. (2008). Assembling an arsenal, the scorpion way. BMC Evol. Biol..

[37] Tamura K., Dudley J., Nei M., Kumar S. (2007). MEGA4: Molecular Evolutionary Genetics Analysis (MEGA) software version 4.0. Mol. Biol. Evol..

[38] Lancelin J.M., Foray M.F., Poncin M., Hollecker M., Marion D. (1994). Proteinase inhibitor homologues as potassium channel blockers. Nat. Struct. Biol..

[39] Zelepuga E.A., Tabakmakher V.M., Chausova V.E., Monastyrnaya M.M., Isaeva M.P., Kozlovskaya E.P. (2012). Interaction of sea anemone *Heteractis crispa* Kunitz type polypeptides with pain vanilloid receptor TRPV1: *In silico* investigation. Russ. J. Bioorg. Chem..

[40] Helland R., Otlewski J., Sundheim O., Dadlez M., Smalås A.O. (1999). The crystal structures of the complexes between bovine beta-trypsin and ten P1 variants of BPTI. J. Mol. Biol..

[41] Vincent J.-P., Lazdunski M. (1972). Trypsin-pancreatic trypsin inhibitor association. Dynamics of the interaction and role of disulfide bridges. Biochemistry.

[42] Delaria K.A., Muller D.K., Marlor C.W., Brown J.E., Das R.C., Roczniak S.O., Tamburini P.P. (1997). Characterization of placental bikunin, a novel human serine protease inhibitor. J. Biol. Chem..

[43] Czapinska H., Helland R., Smalås A.O., Otlewski J. (2004). Crystal structures of five bovine chymotrypsin complexes with P1 BPTI variants. J. Mol. Biol..

[44] Krowarsch D., Dadlez M., Buczek O., Krokoszynska I., Smalås A.O., Otlewski J. (1999). Interscaffolding additivity: Binding of P1 variants of bovine pancreatic trypsin inhibitor to four serine proteases. J. Mol. Biol..

[45] Wang G.J., Gao C.F., Wei D., Wang C., Ding S.Q. (2009). Acute pancreatitis: Etiology and common pathogenesis. World J. Gastroenterol..

[46] Averianov A.V. (2007). Role of neutrophil elastase in pathogenesis of chronic obstructive pulmonary disease. Cytokines Inflamm..

[47] Antuch W., Berndt D.K., Chávez A.M., Delfín J., Wüthrich K. (1993). The NMR solution structure of a Kunitz-type proteinase inhibitor from the sea anemone *Stichodactyla helianthus*. Eur. J. Biochem..

[48] Schwede T., Kopp J., Guex N., Peitsch M.C. (2003). SWISS-MODEL: An automated protein homology-modeling server. Nucleic Acids Res..

[49] Guex N., Peitsch M.C. (1997). SWISS-MODEL and the Swiss-PdbViewer: An environment for comparative protein modeling. Electrophoresis.

[50] Arnold K., Bordoli L., Kopp J., Schwede T. (2006). The SWISS-MODEL Workspace: A web-based environment for protein structure homology modeling. Bioinformatics.

[51] Rizzo R.C., Jorgensen W.L. (1999). OPLS all-atom model for amines: Resolution of the amine hydration problem. J. Am. Chem. Soc..

[52] Ramachandran G.N., Sasisekharan V. (1968). Conformations of polypeptides and proteins. Adv. Protein Chem..

[53] Laskowski R.A., MacArthur M.W., Moss D.S., Thornton J.M. (1993). PROCHECK—a program to check the stereochemical quality of protein structures. J. Appl. Cryst..

[54] Koradi R., Billeter M., Wüthrich K. (1996). MOLMOL: A program for display and analysis of macromolecular structures. J. Mol. Graph..

[55] Kawamura K., Yamada T., Kurihara K., Tamada T., Kuroki R., Tanaka I., Takahashi H., Niimura N. (2011). X-ray and neutron protein crystallographic analysis of the trypsin-BPTI complex. Acta Crystallogr. D Biol. Crystallogr..

[56] Huber R., Kukla D., Bode W., Schwager P., Bartels K., Deisenhofer J., Steigemann W.  (1974). Structure of the complex formed by bovine trypsin and bovine pancreatic trypsin inhibitor: II. Crystallographic refinement at 1.9 Å resolution. J. Mol. Biol..

[57] Il’ina A., Lipkin A., Barsova E., Issaeva M., Leychenko E., Guzev K., Monastyrnaya M., Lukyanov S., Kozlovskaya E. (2006). Amino acid sequence of RTX-A’s isoform actinoporin from the sea anemone, *Radianthus macrodactylus*. Toxicon.

[58] Saitou N., Nei M. (1987). The neighbor-joining method: A new method for reconstructing phylogenetic trees. Mol. Biol. Evol..

[59] Zuckerkandl E., Pauling L., Bryson V., Vogel H.J. (1965). Evolutionary Divergence and Convergence in Proteins. Evolving Genes and Proteins.

[60] Liman E.R., Tytgat J., Hess P. (1992). Subunit stoichiometry of a mammalian K+ channel determined by construction of multimeric cDNAs. Neuron.

[61] Caterina M.J., Schumacher M.A., Tominaga M., Rosen T.A., Levine J.D., Julius D. (1997). The capsaicin receptor: A heat-activated ion channel in the pain pathway. Nature.

[62] Kozakov D., Brenke R., Comeau S.R., Vajda S. (2006). PIPER: An FFT-based protein docking program with pairwise potentials. Protein.

[63] Marquart M., Walter J., Deisenhofer J., Bode W., Huber R. (1983). The Geometry of the reactive site and of the peptide groups in trypsin, trypsinogen and its complexes with inhibitors. Acta Cryst. Sect. B.

[64] Scheidig A.J., Hynes T.R., Pelletier L.A., Wells J.A., Kossiakoff A.A. (1997). Crystal structures of bovine chymotrypsin and trypsin complexed to the inhibitor domain of Alzheimer's amyloid beta-protein precursor (APPI) and basic pancreatic trypsin inhibitor (BPTI): Engineering of inhibitors with altered specificities. Protein Sci..

[65] Meireles L.M.C., Dömling A.S., Camacho C.J. (2010). ANCHOR: A web server and database for analysis of protein-protein interaction binding pockets for drug discovery. Nucleic Acids Res..

[66] Krissinel E., Henrick K. (2007). Inference of macromolecular assemblies from crystalline state. J. Mol. Biol..

[67] Krissinel E. (2010). Crystal contacts as nature’s docking solutions. J. Comput. Chem..

[68] Camacho C.J., Zhang C. (2005). FastContact: Rapid estimate of contact and binding free energies. Bioinformatics.

